# Supplementary material for: Phage Display-Based Homing Peptide-Daunomycin Conjugates for Selective Drug Targeting to PANC-1 Pancreatic Cancer
Source: Pharmaceutics. 2020 Jun 22;12(6):576. doi: 10.3390/pharmaceutics12060576 (PMC7355684; doi:10.3390/pharmaceutics12060576)
Supplement: Supplementary file 1 [file pharmaceutics-12-00576-s001.pdf]

Supplementary information

Phage display-based homing peptide - daunomycin conjugates for selective drug targeting to PANC-1 Pancreatic Cancer

Levente E. Dókus[1,2], Eszter Lajkó[3], Ivan Ranđelović[4], Diána Mező[3], Gitta Schlosser[1,2], László Kőhidai[3], József Tóvári[4] and Gábor Mező[1,2*]

[1] MTA-ELTE Research Group of Peptide Chemistry, Hungarian Academy of Sciences, Budapest, Hungary (doehabt@caesar.elte.hu)

[2] Eötvös Loránd University, Faculty of Sciences, Institute of Chemistry, Budapest, Hungary

[3] Department of Genetics, Cell- and Immunobiology, Semmelweis University, Budapest, Hungary

[4] National Institute of Oncology, Department of Experimental Pharmacology, Budapest, Hungary

[*] Correspondence: gmezo@elte.hu, Tel.: +36 1 372-2500/1430

Table S1. *In vitro* cytotoxic effect of the prepared conjugates on various cell lines

Code	Compounds	Viability[a] (%) at 10^{-5} M concentration, after 72 h incubation		
		Colo-205	A2058	EBC-1
1	*Dau=Aoa*-SKAAKN-*OH*	50.7 ± 1.6	61.5 ± 2.7	77.8 ± 1.0
2	*Dau=Aoa*-KSKAAKN-*OH*	51.0 ± 2.5	63.5 ± 4.6	90.2 ± 3.0
3	*Dau=Aoa*-GFLG-KSKAAKN-*OH*	59.3 ± 3.6	79.4 ± 17.6	76.2 ± 13.7
4	*Dau=Aoa*-GFLG-K(*Dau=Aoa*)SKAAKN-*OH*	19.8 ± 0.5	29.8 ± 4.5	32.2 ± 4.2
5	*Dau=Aoa*-GFLG-K(*Dau=Aoa*-GFLG)SKAAKN-*OH*	45.8 ± 2.6	61.9 ± 11.6	57.6 ± 11.1

[a] Fluorescence intensity data of the treated cells are normalized to the fluorescence intensity data values of the control wells and expressed as percentages. Data are given as mean values ± standard deviation (SD), (n=3).

Table S2. Relative fluorescence intensity results of binding and cellular uptake measurements on PANC-1 cells

Code	Compounds	Relative fluorescence intensity (GeoMean values)[a]	
		Binding	Cellular uptake
	Daunomycin	72.057 ± 3.907	702.048 ± 84.340
1	*Dau=Aoa*-SKAAKN-*OH*	0.086 ± 0.173	1.630 ± 0.356
2	*Dau=Aoa*-KSKAAKN-*OH*	0.830 ± 0.157	5.228 ± 0.257
3	*Dau=Aoa*-GFLG-KSKAAKN-*OH*	0.012 ± 0.011	0.459 ± 0.189
4	*Dau=Aoa*-GFLG-K(*Dau=Aoa*)SKAAKN-*OH*	0.463 ± 0.147	7.666 ± 1.147
5	*Dau=Aoa*-GFLG-K(*Dau=Aoa*-GFLG)SKAAKN-*OH*	0.071 ± 0.082	2.779 ± 0.575

[a] Binding and cellular uptake of the compounds were studied at 10^{-5} M concentration. GeoMean value (geometric mean channel value) is a dimensionless value and refers to the relative fluorescence intensity. Data shown are mean of 2 parallels ± SD.

Analytical chromatograms of compounds:

Analytical RP-HPLC was performed on a Waters Symmetry (WAT 045905) C18 column (150x4.6mm I.D.) with 5 μm silica (100 Å pore size) as a stationary phase. A linear gradient elution was developed: 0 min 0% B; 2 min 0% B; 22 min 90% B with eluent A (0.1% TFA in water) and eluent B (0.1% TFA in acetonitrile-water (80: 20, v/v)). A flow rate of 1 mL/min was used at ambient temperature. Samples were applied dissolved in eluent A and 20 μl was injected. Peaks were detected at λ = 220 nm.

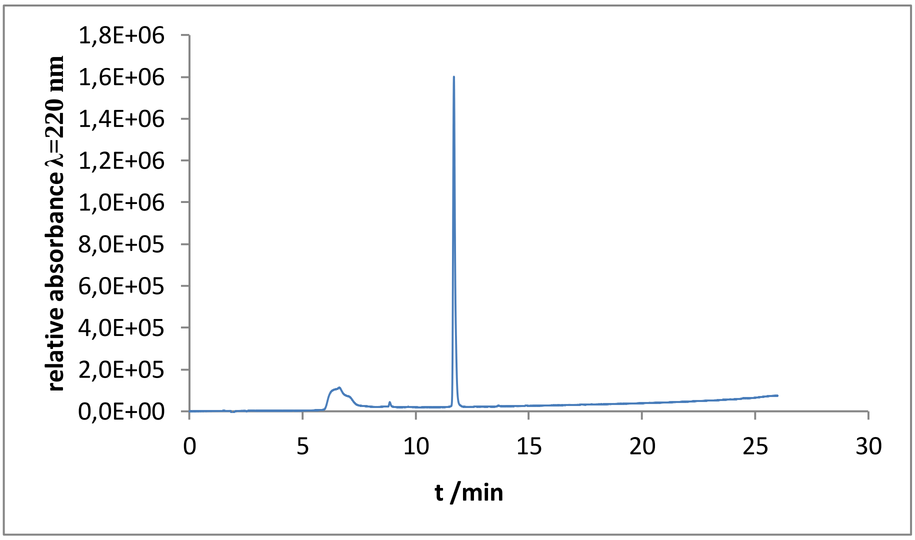

Figure S1. Analytical chromatogram of compound **1** (*Dau=Aoa*-SKAAKN-*OH*)

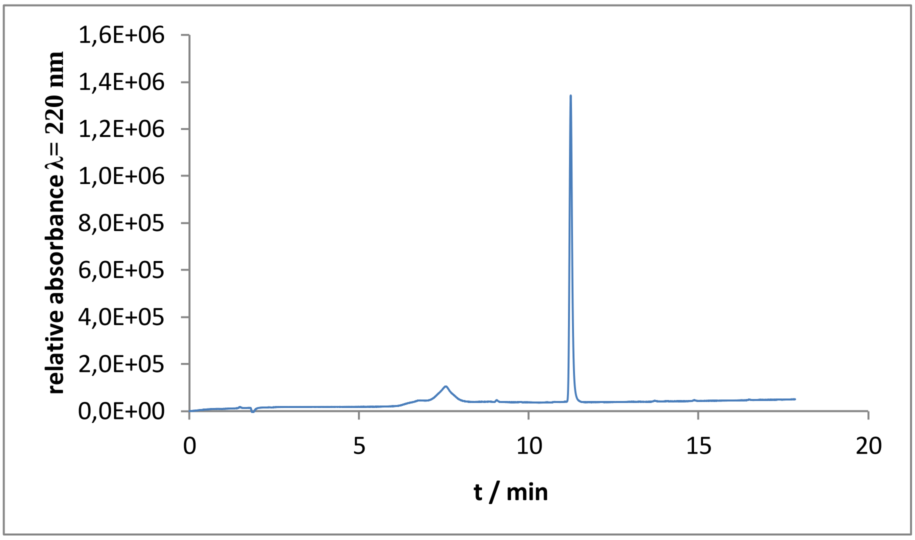

Figure S2. Analytical chromatogram of compound **2** (*Dau*=*Aoa*-KSKAAKN-*OH*)

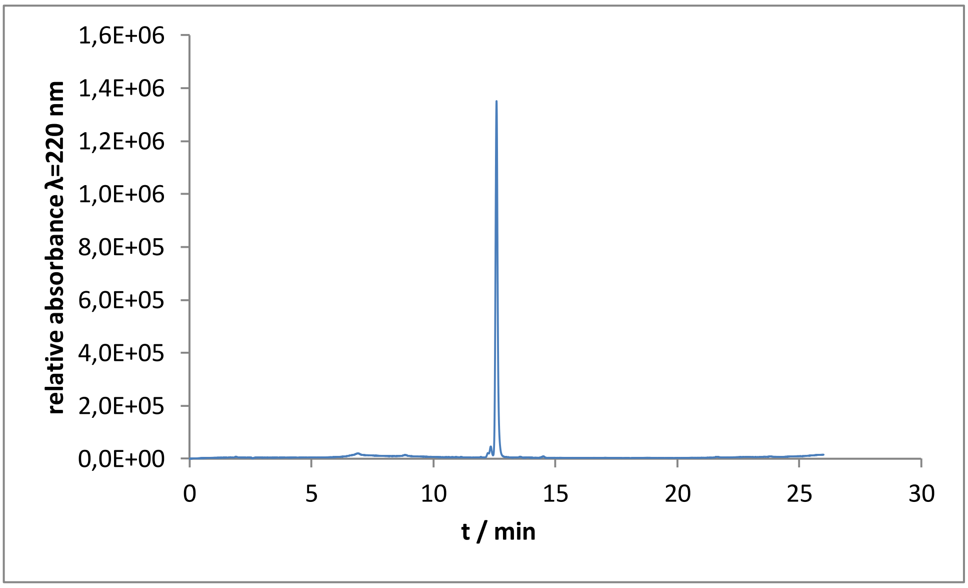

Figure S3. Analytical chromatogram of compound **3** (*Dau*=*Aoa*-GFLGKSKAAKN-*OH*)

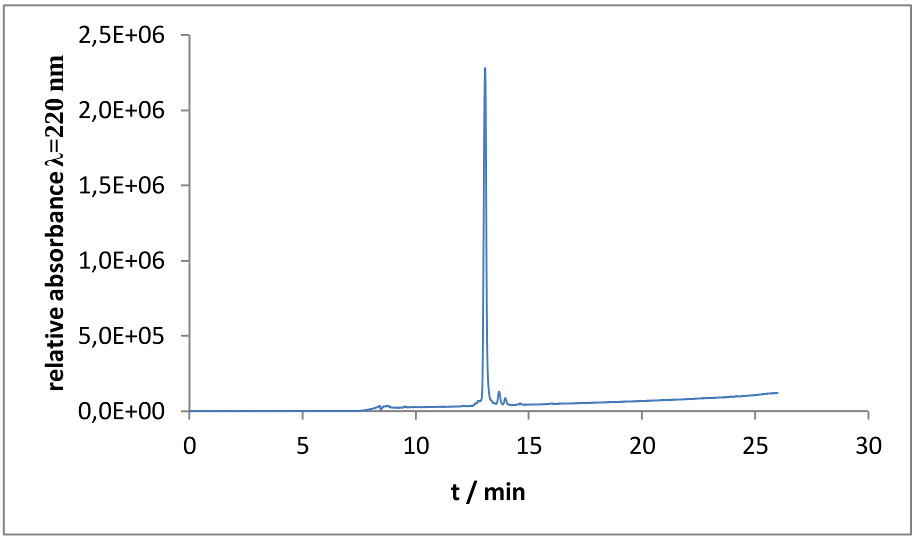

Figure S4. Analytical chromatogram of compound **4** (*Dau=Aoa*-GFLGK(*Dau=Aoa*)SKAAKN-*OH*)

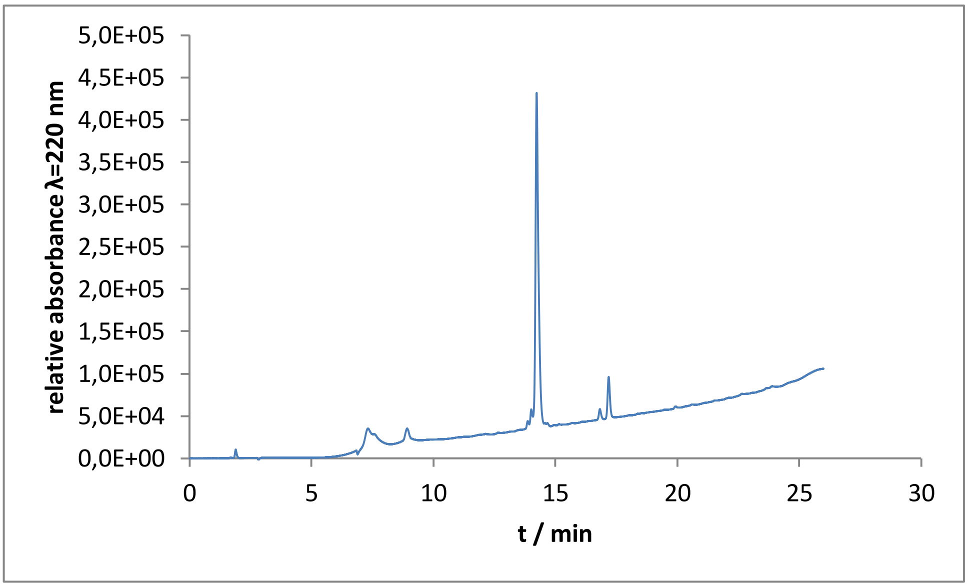

Figure S5. Analytical chromatogram of compound (*Dau=Aoa*-GFLG)K(*Dau=Aoa*-GFLG)SKAAKN-*OH*)

Mass spectrometry

The identification of the peptide analogues and conjugates was achieved by electrospray ionization mass spectrometry (ESI-MS) on a Bruker Daltonics Esquire 3000 Plus (Bremen, Germany) ion trap mass spectrometer, operating in continuous sample injection at 4 μL/min flow rate. Samples were dissolved in ACN-water (50:50 v/v%) mixture containing 0.1 v/v% AcOH. Mass spectra were recorded in positive ion mode in the *m*/*z* 50-2000 range.

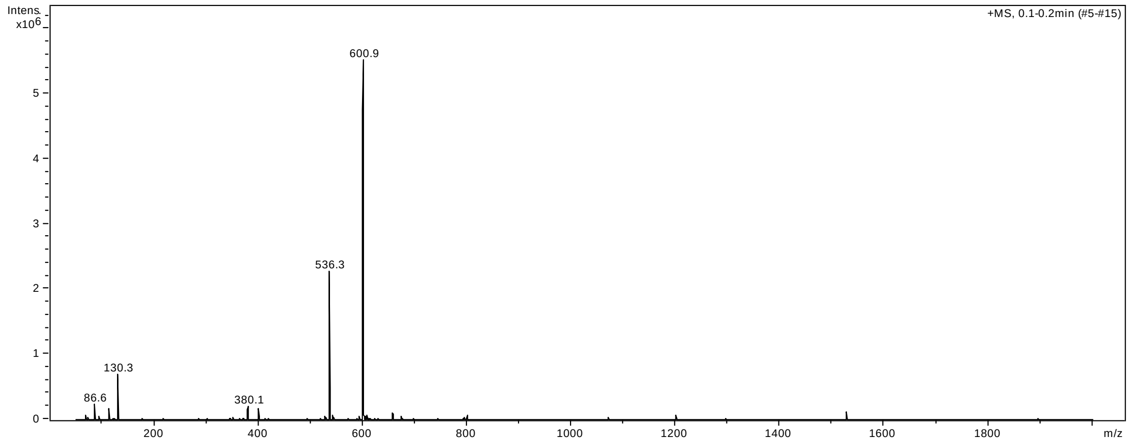

Figure S6. Mass spectra of compound **1**

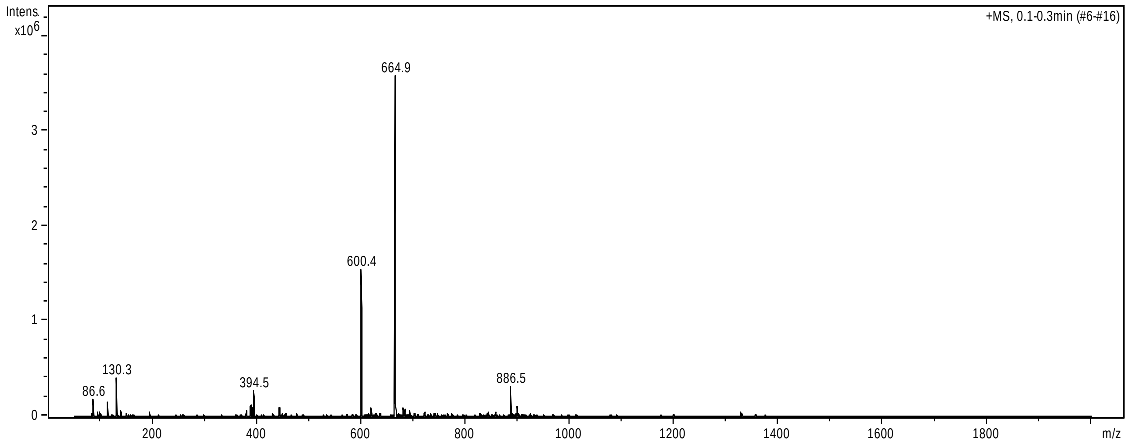

Figure S7. Mass spectra of compound **2**

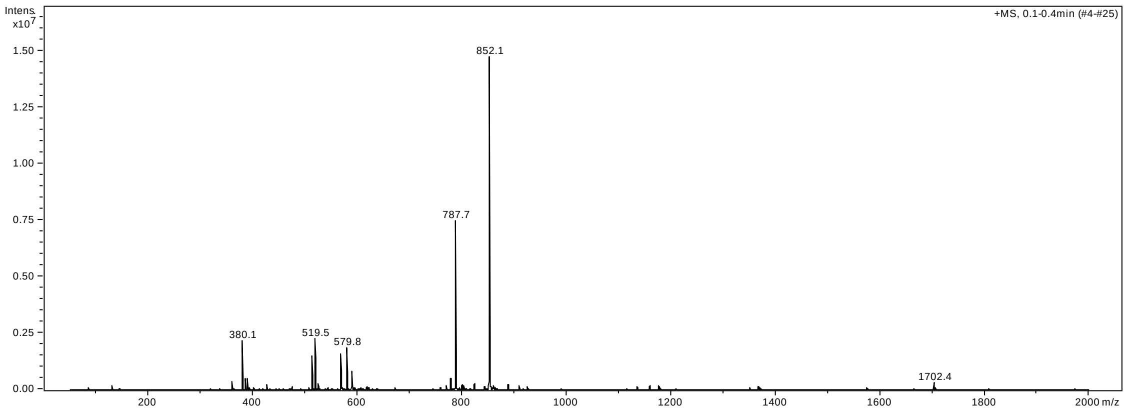

Figure S8. Mass spectra of compound **3**

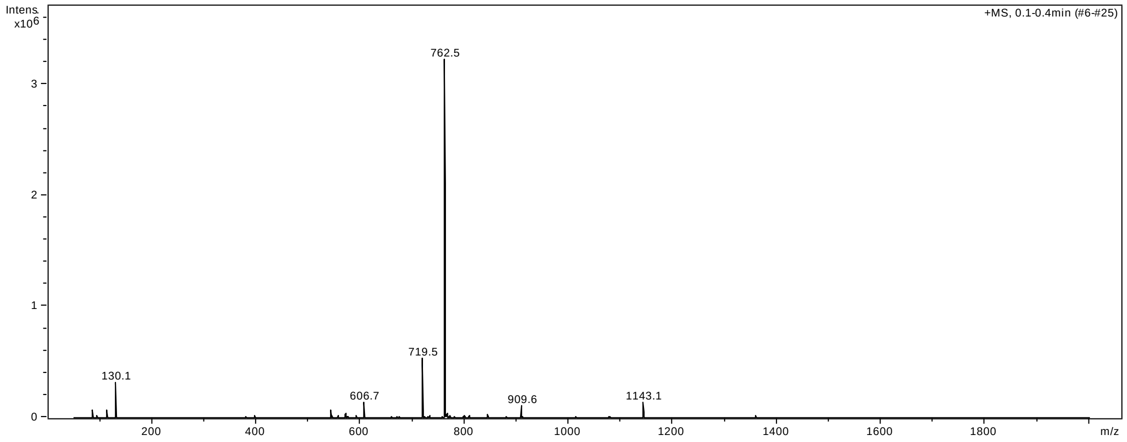

Figure S9. Mass spectra of compound **4**

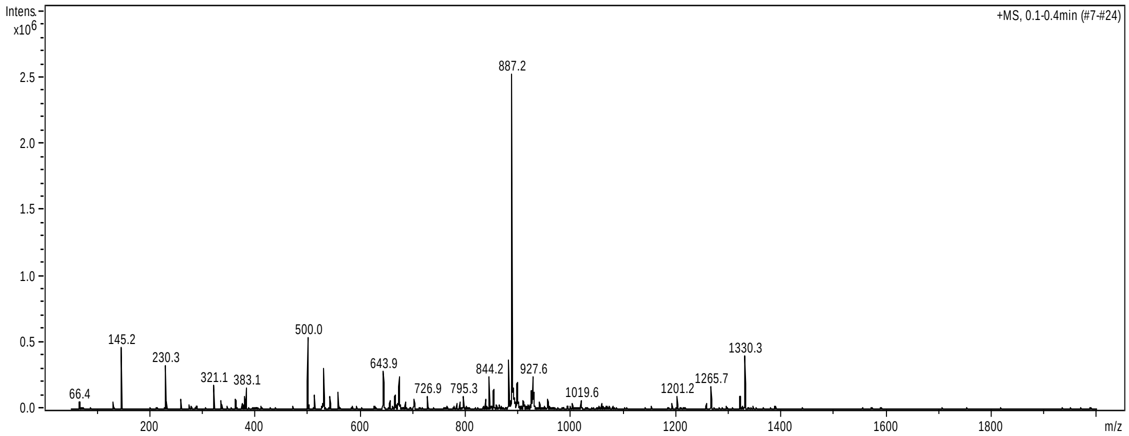

Figure S10: Mass spectra of compound **5**

Chemical characterization of identified fragments of the various conjugates under the enzymatic degradation

Table S3. Chemical characterization of identified fragments of conjugate **1**

**Product of the degradation**	**R_t (min.)**	**$M_{(cal.)}$**	**$M_{(meas.)}$**
Dau=Aoa-S-*OH*	11.17	687.2276	687.2274
Dau=Aoa-SK-*OH*	9.99	815.3225	815.3217

Table S4. Chemical characterization of identified fragments of conjugate **2**

**Product of the degradation**	**R_t (min.)**	**$M_{(cal.)}$**	**$M_{(meas.)}$**
Dau=Aoa-K-*OH*	11.4	728.2905	728.2900
Dau=Aoa-KS-*OH*	12.5	815.3225	815.3223
Dau=Aoa-KSK-*OH*	13.1	943.4175	943.4171

Table S5. Chemical characterization of identified fragments of conjugate **3**

Product of the degradation	R_t (min.)	$M_{(cal.)}$	$M_{(meas.)}$
Dau=Aoa-G-*OH*	11.4	657.2170	657.2168
Dau=Aoa-GF-*OH*	12.5	804.2854	804.2855
Dau=Aoa-GFL-*OH*	13.1	917.3695	917.3690
Dau=Aoa-GFLGK-*OH*	11.9	1102.4859	1102.4851
Dau=Aoa-GFLGKSK-*OH*	10.5	1317.6129	1317.6122
Dau=Aoa-GFLGKSKAA-*OH*	10.5	1459.6871	1459.6864
Dau=Aoa-GFLGKSKAAKN-*OH*	9.9	1701.8250	1701.8237

Table S6. Chemical characterization of identified fragments of conjugate **4**

Product of the degradation	R_t (min.)	$M_{(cal.)}$	$M_{(meas.)}$
Dau=Aoa-G-*OH*	11.4	657.2170	657.2170
Dau=Aoa-GF-*OH*	12.5	804.2854	804.2852
Dau=Aoa-GFL-*OH*	13.1	917.3695	917.3692
H-K*(Dau=Aoa)-OH*	10.6	728,2905	728.2896
H-GK*(Dau=Aoa)-OH*	10.0	785.3120	785.3118
H-K*(Dau=Aoa)*S-*OH*	10.0	815.3225	815.3222
H-K*(Dau=Aoa)*SK-*OH*	9.1	943.4175	943.4163
H-K*(Dau=Aoa)*SKAAKN-*OH*	8.3	1327.6246	1327.6273
Dau=Aoa-GFLGK(*Dau=Aoa*)SK-*OH*	11.0	1899.7978	1899.7974
Dau=Aoa-GFLGK(*Dau=Aoa*)SKAAKN-*OH*	10.3	2284.0100	2284.0069

Table S7. Chemical characterization of identified fragments of conjugate **5**

Product of the degradation	R_t (min.)	$M_{(cal.)}$	$M_{(meas.)}$
Dau=Aoa-G-*OH*	11.4	657.2170	657.2168
Dau=Aoa-GF-*OH*	12.5	804.2854	804.2855
Dau=Aoa-GFL-*OH*	13.1	917.3695	917.3690
H-K(*Dau=Aoa*-GFLG)-*OH*	11.9	1102.4859	1102.4851
H-K(*Dau=Aoa*-GFLG)SK-*OH*	10.5	1317.6129	1317.6122
H-K(*Dau=Aoa*-GFLG)SKAA-*OH*	10.5	1459.6871	1459.6864
H-K(*Dau=Aoa*-GFLG)SKAAKN-*OH*	9.9	1701.8200	1701.8239
Dau=Aoa-GFLGK(*Dau=Aoa*-GFLG)SKAAKN-*OH*	11.1	2658.2054	2658.2037
